# Rapid and Routine Molecular Typing Using Multiplex Polymerase Chain Reaction and MinION Sequencer

**DOI:** 10.3389/fmicb.2022.875347

**Published:** 2022-03-29

**Authors:** Yu-Chieh Liao, Han-Chieh Wu, Ci-Hong Liou, Tsai-Ling Yang Lauderdale, I-Wen Huang, Jui-Fen Lai, Feng-Jui Chen

**Affiliations:** ^1^Institute of Population Health Sciences, National Health Research Institutes, Zhunan Town, Miaoli County, Taiwan; ^2^National Institute of Infectious Diseases and Vaccinology, National Health Research Institutes, Zhunan Town, Miaoli County, Taiwan; ^3^Department of Biological Science and Technology, National Yang Ming Chiao Tung University, Hsinchu, Taiwan

**Keywords:** nanopore sequencing, molecular typing, multiplex polymerase chain reaction, multilocus sequence typing, MinION

## Abstract

Molecular typing is an essential tool that has been extensively applied in laboratories as well as in clinical settings. Next-generation sequencing technologies promise high-throughput and cost-effective molecular applications; however, the accessibility of these technologies is limited due to the high capital cost. Oxford Nanopore Technologies (ONT) offers a MinION device with the advantages of real-time data analysis, rapid library preparation, and low cost per test. However, the advantages of the MinION device are often overshadowed by its lower raw accuracy. Herein, we present a concise multilocus sequence typing protocol of *Staphylococcus aureus* using multiplex polymerase chain reaction and Rapid Barcoding Kit for barcoding and MinION device for sequencing. Moreover, to clarify the effects of carryover DNA on tasks that require high sequence accuracy, we used the MinION flow cell in successive runs of washing and reusing. Our results revealed that the MinION flow cell could achieve accurate typing of a total of 467 samples with 3,269 kilobase-long genes within a total of 5 runs. This thus demonstrates the effectiveness of a portable nanopore MinION sequencer in providing accurate, rapid, and routine molecular typing.

## Introduction

Molecular diagnostics is widely used in clinical microbiology for routine detection and epidemiological analysis of infectious microorganisms (Chen et al., 2018). The invention of polymerase chain reaction (PCR) has led to remarkable developments in clinical molecular diagnostics because the use of PCR-based technologies requires relatively simple instrumentation and only small amounts of biological material (Maheaswari et al., 2016). PCR-based molecular diagnostic methods are important in studies of infectious diseases. For examples, 16S rRNA gene/internal transcribed spacer region sequencing is a well-established method for bacterial and fungal identification (Raja et al., 2017; Peker et al., 2019), and multilocus sequence typing (MLST) has become a commonly applied technique in molecular evolution studies of numerous microbial species (Jolley et al., 2018). Sanger sequencing is commonly used to obtain sequences of interest; however, the cost of hundreds of samples is prohibitive (Kircher and Kelso, 2010). Next-generation sequencing (NGS) technologies (e.g., PacBio and Illumina) have been used to achieve high-throughput and cost-effective molecular diagnostics (Chen et al., 2015, 2018; Perez-Losada et al., 2018; Zhang et al., 2018; Peker et al., 2019), which has greatly affected clinical microbiology. However, the large costs associated with installing NGS instrumentation limit the accessibility of rapid and routine molecular typing in small- to medium-sized laboratories.

Oxford Nanopore Technologies (ONT) currently offer an inexpensive, pocket-sized MinION device that produces long sequences; however, the raw reads from this device are of lower accuracy in comparison with Illumina platform (Lin et al., 2021). In conjunction with consensus sequence generation and homopolymer correction, accurate molecular sequences can be obtained through nanopore sequencing (Liou et al., 2020). MinION sequencer provides the advantages of real-time data analysis, low capital cost, and highly accurate consensus sequence generation, all of which are adequately suited to the constraints of clinical settings (Sheka et al., 2021). Therefore, MinION sequencer has been used in numerous applications of clinical microbiology and infectious diagnostics (Ma et al., 2013; Benitez-Paez et al., 2016; Liou et al., 2020; Baldan et al., 2021; Ben et al., 2021; Ferreira et al., 2021; Sheka et al., 2021; Snell et al., 2021; Urban et al., 2021). However, the sample size for a MinION flow cell cannot exceed 96 due to the limitations of the barcoding kits. Although several studies have used tailored primers (Currin et al., 2019) or proposed dual-barcode systems (Liou et al., 2020) to address the problem of sample size, a requirement of additional efforts or costs remains inevitable. Furthermore, although ONT provides a Flow Cell Wash Kit, the influence of repeated washing and use of a MinION flow cell on the accuracy of a consensus sequence has yet to be comprehensively studied. With the recent release of the Rapid Barcoding Kit 96 (SQK-RBK110.96, released on March 2021) and the increasing demand for routine molecular diagnostics, evaluating the capabilities of a single MinION flow cell for accurate, timely, and routine molecular typing has become imperative.

Accordingly, in this study, we proposed a rapid protocol entailing the use of multiplex PCR of seven housekeeping genes and rapid barcoding of 392 *Staphylococcus aureus* isolates in conjunction with a MinION flow cell for sequencing to obtain a total of 3,269 kilobase-long consensus sequences. In addition to using Krocus (Page and Keane, 2018) for rapid MLST of *S. aureus*, we implemented nanoMLST2, which was modified from our previously proposed nanoMLST (Liou et al., 2020), for consensus sequence generation. Sixteen new alleles were identified by nanoMLST2 and validated with Sanger sequencing. The study results suggest that MinION nanopore sequencing of multiplex PCR amplicons could be a cost-effective method for rapid and routine molecular typing.

## Materials and Methods

### Bacterial Isolates and DNA Extraction

A total of 392 *S. aureus* isolates were used in this study (designated as Sau 1–392). The isolates were collected from the Taiwan Surveillance of Antimicrobial Resistance program, a national surveillance program in Taiwan (Ho et al., 1999). Bacterial DNA templates from pure cultures were prepared using DNAzol Direct (Molecular Research Center, Inc. Cincinnati, OH, United States), according to the manufacturer’s instructions. Of the 392 *S. aureus* isolates, 88 had been subjected to MinION nanopore sequencing to determine sequencing types (STs) in a previous study of 96 isolates (Liou et al., 2020) and were used in the second and the fourth runs as references to validate the accuracy of the workflow. Furthermore, 50 of these 88 isolates had DNA templates (i.e., 350 alleles), and two alleles, namely *pta*_664 and *glpF*_732, had been subjected to Sanger sequencing (Liou et al., 2020).

### Multiplex PCR

Seven housekeeping genes were subjected to multiplex PCR using the Thermo Scientific Phusion High-fidelity DNA Polymerase kit (Thermo Fisher Scientific, Waltham, MA, United States) in a total volume of 25 μl (5 μl of 5× HF buffer, 2 μl of 2.5 mM dNTP, 10 μl of primer mix, 0.25 μl of Phusion enzyme, 1 μl of DNA template, and 6.75 μl of nuclease-free water). The primer sequences are listed in Supplementary Table. The primer mix included 10 μM each of forward and reverse primers of carbamate kinase (*arcC*), shikimate dehydrogenase (*aroE*), glycerol kinase (*glpF*), guanylate kinase (*gmk*), phosphate acetyltransferase (*pta*), triosephosphate isomerase (*tpi*), and acetyl coenzyme A acetyltransferase (*yqiL*) in a balanced ratio. The PCR program was set as follows: initial denaturation at 98°C for 30 s followed by 35 cycles of denaturation at 98°C for 10 s, annealing at 65°C for 30 s, and extension at 72°C for 1 min; and then a single final extension at 72°C for 10 min.

### Library Preparation and Sequencing

The newly released Rapid Barcoding Kit 96 (SQK-RBK110.96) was used for the rapid barcoding of the 96 samples. Each sample was mixed with 5 μl of multiplexing PCR product, 2.5 μl of nuclease-free water, and 2.5 μl of one rapid barcode. The mixture was incubated at 30°C for 2 min, followed by incubation at 80°C for 2 min. All 96 barcoded DNA samples were pooled, and 120 μl of the pooled DNA was sampled and mixed with an equal volume of solid phase reversible immobilization beads (SPRI). After 5 min of incubation on a hula mixer, the barcoded DNA was cleaned twice with 240 μl of 80% ethanol and eluted with 30 μl of elution buffer (EB). An aliquot of 800 ng of barcoded DNA was used to make up a total volume of 11 μl with EB. One microliter of rapid adaptor F was added to the barcoded DNA, and the mixture was incubated at room temperature for 10 min. A pre-sequencing mix (PSM) was prepared by adding 37.5 μl of Sequencing Buffer II and 25.5 μl of loading beads to a 12 μl DNA library. The PSM was loaded *via* the SpotON port into a primed Flow cell (FLO-106MIN) for sequencing. Basecalling and de-multiplexing were performed in real time through MinKNOW GUI (v4.3.4) implemented with GPU Guppy (v5.0.11) on a desktop PC with an NVIDIA RTX 3090 graphics card with 24-GB RAM to produce high-accuracy reads in FASTQ format. The default output set for MinKNOW was a FASTQ file containing 4,000 reads.

### Data Analysis

A sequencing run was stopped when individual FASTQ output files were obtained for each barcoded sample; this is because the derivation of such output files suggested more than 4,000 reads had been obtained for the sample. The FASTQ files obtained for each sample were collected and analyzed using Krocus 1.0.1 (Page and Keane, 2018) with a prepared directory named “Staphylococcus_aureus” which contained *S. aureus* MLST alleles and allelic profiles downloaded from PubMLST (Jolley et al., 2018). For each sample, consensus sequences were generated using Medaka v1.4.3^1^ along with the FASTQ file and the reference sequences of seven housekeeping genes of *S. aureus* NCTC8325 (Liou et al., 2020). Samples with gene reads fewer than 40 were identified and labeled as “LSD (low sequencing depth)” by aligning the sequencing reads against the reference sequences using Minimap2 (v2.20; Li, 2018). Homopolymer errors registered for the consensus sequences were corrected, if necessary, to assign MLST alleles and to profile sequence types using a modified script, namely runtyping.py, in nanoMLST (Liou et al., 2020); this updated workflow was denoted as nanoMLST2, and it is available at https://github.com/jade-nhri/nanoMLST2.

### Flow Cell Wash and Reuse

When 4,000 reads had been collected for each of the 96 samples, the sequencing experiment was stopped. The flow cell was left in the device. A flow cell wash mix was prepared by mixing 398 μl of wash diluent (DIL) and 2 μl of wash mix (WMX) from the Flow Cell Wash Kit (EXP-WSH004); this mixture was then loaded into the flow cell through the priming port. After 1 h of incubation at room temperature, 500 μl of storage buffer (S) was added through the priming port. The priming port was then closed to allow for the removal of all fluid from the waste channel through the waste port. The washed flow cell was stored at 4°C for reuse. The same cell was used five times on a total of 480 samples (96 samples per run, a total of 392 isolates). Notably, 88 PCR amplicons in the fourth run were aliquots from the second run but were barcoded with different barcodes. Another flow cell was used to ensure the reproducibility of this study.

### Sanger Sequencing

Allele types, determined through Krocus and nanoMLST2 were compared to identify inconsistencies. The inconsistent alleles were further subjected to Sanger sequencing with conventional *S. aureus* MLST primers (Jolley et al., 2018).

## Results and Discussion

### Rapid Library Preparation and Real-Time MinION Sequencing

**Figure 1:**
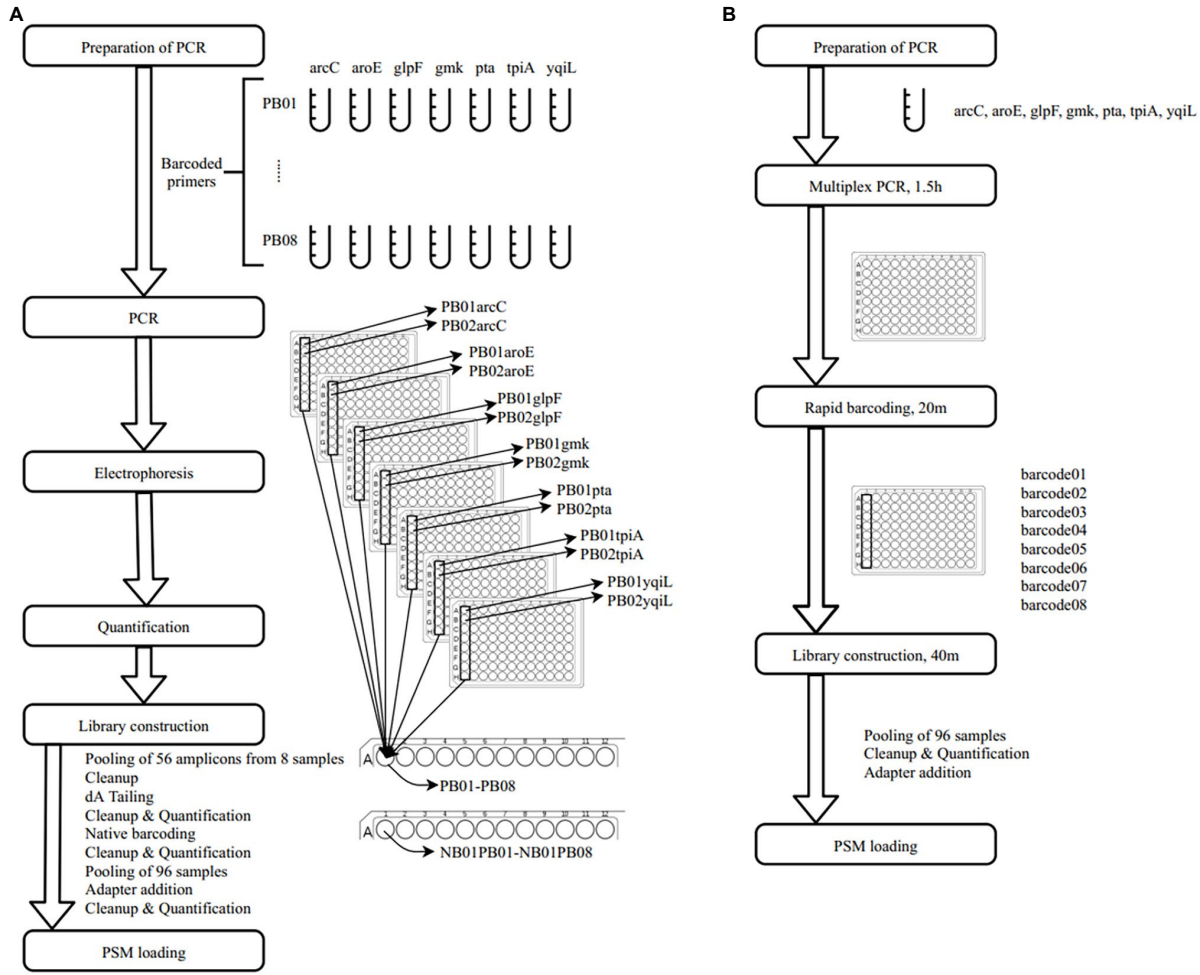
Schematic workflows of **(A)** dual-barcode system (Liou et al., 2020) and **(B)** rapid multiplex polymerase chain reaction (PCR) and barcoding protocol.

**Figure 2:**
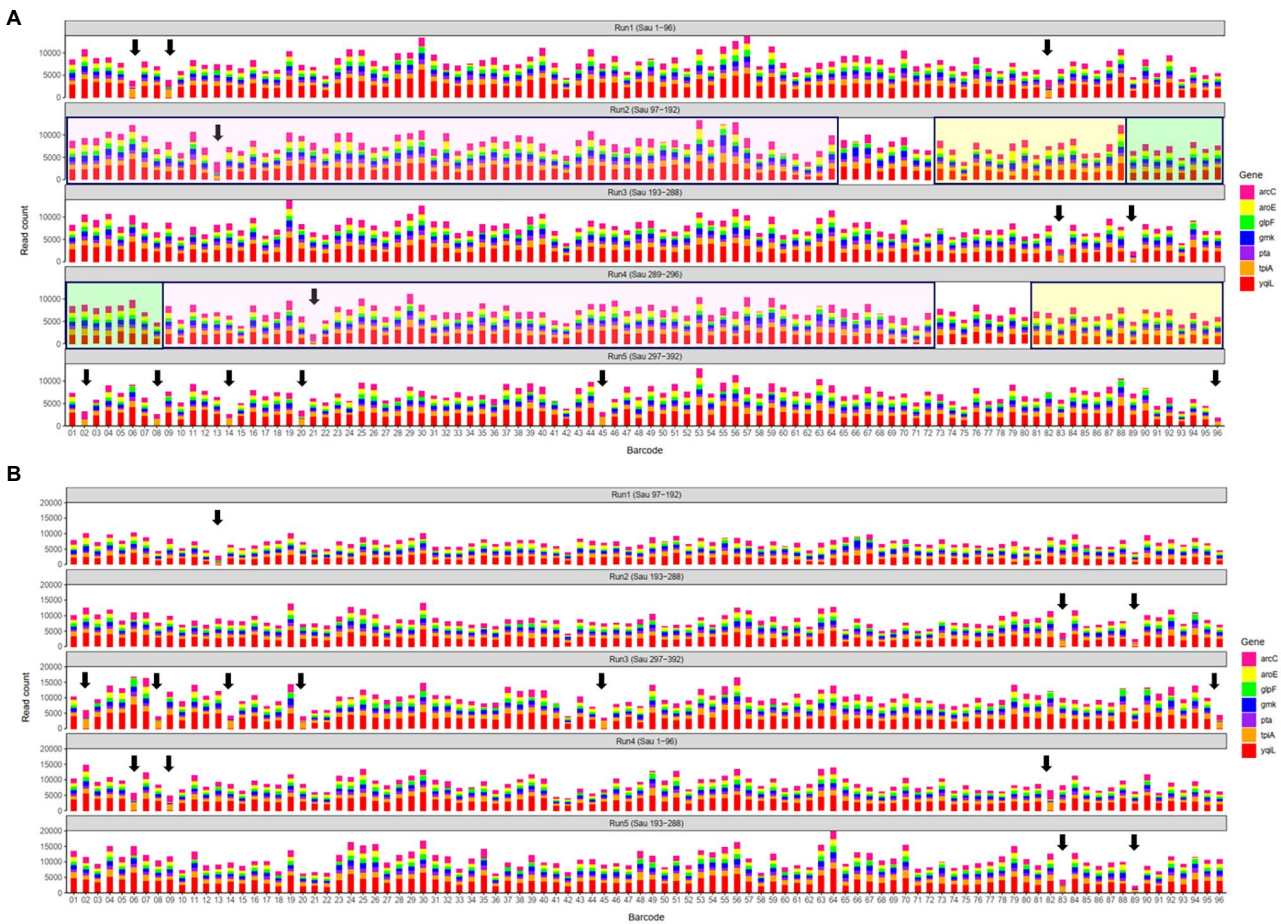
Distributions of sequencing reads on the first flow cell **(A)** and the second flow cell **(B)**. Arrows indicate samples labeled as low sequencing depth (LSD). In **(A)**, 88 polymerase chain reaction (PCR) products in Run4 highlighted with background colors to indicate aliquots of amplicons in Run2 that were rapid barcoded with different barcodes; the other 8 PCR products in Run4 were from 8 isolates (Sau 289–296). Run1–Run5 in **(B)** containing identical PCR products in Run2, Run3, Run5, Run1, and Run3 in **(A)**, respectively.

**Figure 3:**
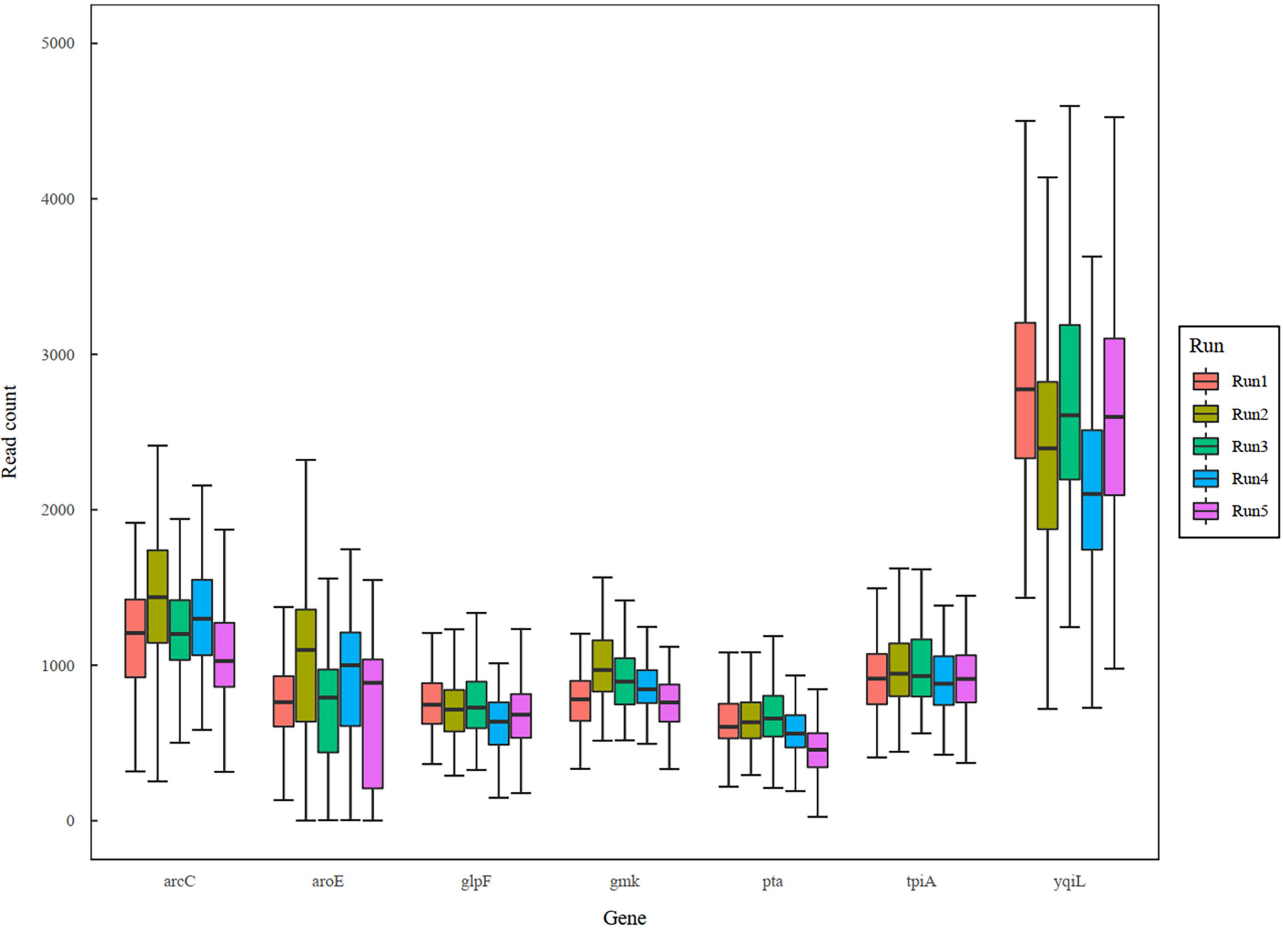
Boxplot of sequencing reads across genes and runs.

In a previous study, a dual-barcoding system was established to multiplex 96 *S. aureus* isolates for seven housekeeping genes using 12 native barcodes in combination with 8 × 7 pairs of primers (Liou et al., 2020). The throughput of MinION nanopore sequencing, with careful electrophoresis and quantification processes, was estimated to be sufficient for 1,000 samples (Liou et al., 2020). However, the study used a labor-intensive and time-consuming process (Figure 1A); furthermore, ordering 96 × 7 pairs of primers solely for *S. aureus* MLST is impractical and cost prohibitive. Accordingly, we devised a new process involving multiplex PCR and the rapid barcoding of 96 isolates based on the newly released Rapid Barcoding Kit 96 (SQK-RBK110.96, released in March 2021) in 3 h (Figure 1B), as a preparation for MinION nanopore sequencing. A sequencing run was conducted with the objective of achieving 4,000 reads per sample; the five successive runs required 3.6, 3.9, 5, 9.8, and over 48 h (Table 1). A reduced pass rate was observed, which might have been engendered by the impairment of the integrity of the reused pores; the reduced pass rate along with the decrease in available pores may have increased unclassified rates and run times. Nevertheless, sufficient reads were available for the molecular typing of the samples in all five runs. As illustrated in Figure 1B, to simplify the process, the quantification steps were omitted before the pooling of the 96 samples. This rapid protocol requires minimal effort for quantification. To execute PCR, a DNA template (1 μl) was applied through a single-tube multiplex PCR assay (a total volume of 25 μl containing seven pairs of primers for *S. aureus* MLST). To achieve rapid barcoding, a 5 μl multiplex PCR product was mixed with a barcode. After the pooled DNA was sampled, only cleanup and quantification were required prior to the preparation of a PSM. Despite this simplification, among the 392 multiplex PCR products from the five runs, an extremely high (380/392 = 97%) success rate was observed for the amplification process in our protocol, with only 12 samples being labeled as low sequencing depth (LSD; exclusive of 88 samples—barcode01-barcode72 and barcode81-barcode96—in Run4 of Figure 2A); this can be attributed to the presence of samples with gene reads fewer than 40. Notably, 88 PCR products in Run4 (highlighted with background colors in Figure 2A) were aliquots of amplicons in Run2 but were barcoded with different barcodes. Among the 88 PCR products, 50 had been previously Sanger sequenced (Liou et al., 2020). They were used as references to evaluate whether carryover reads influence typing accuracy. Besides, the rapid protocol provided stable read counts for nearly every gene, except for those with high amounts of *yqi* (Figure 3). Although 88 PCR products in Run4 were aliquots of amplicons in Run2 but with different barcodes, the read counts of the 88 counterparts in Run2 and Run4 were not correlated (*R*^2^ = 0.017); conversely, the read counts of the barcoded samples between runs were moderately correlated (*R*^2^ for the correlation between R1 and Run2–Run5: from 0.289 to 0.555 and from 0.355 to 0.460 for the two flow cells, respectively). This suggests that some barcodes tend to have high or low read counts; for example, barcode30 and barcode56 had high read counts, but barcode42 and barcode89 had low read counts. This may be useful for executing ratio adjustment in order to obtain even distributions. Future research should focus on the refinement of multiplex primers to provide a narrow distribution of read counts among all genes. Our rapid protocol successfully amplified 380 out of 392 *S. aureus* isolates and required less than 3 h of library preparation per 96 samples, indicating that this protocol can facilitate rapid and routine molecular typing and can be easily adapted to different applications.

**Table 1:** Summary of MinION nanopore sequencing results.

Run	Available pores	Run time	Read counts	Passed reads	Passed (%)	Barcoded reads	Unclassified (%)	Average barcoded reads
Run1	830	3 h 39 m 9 s	934,114	829,160	88.8	786,375	5.2	8191.4 ± 1963.2
Run2	572	3 h 56 m 24 s	983,673	849,178	86.3	803,037	5.4	8365.0 ± 1911.2
Run3	510	5 h 3 m 47 s	1,021,596	843,838	82.6	795,398	5.7	8285.4 ± 1932.1
Run4	379	9 h 47 m 53 s	998,293	765,018	76.6	716,831	6.3	7467.0 ± 1552.1
Run5	325	48 h 2 m 24 s	1,092,802	764,021	69.9	707,887	7.3	7373.8 ± 1828.7

### Rapid Sequence Typing Using Krocus

Through real-time basecalling and de-multiplexing in MinKnow, FASTQ files of the 96 barcoded amplicons were produced in only 4 h in the first two runs. Through parallel processing, Krocus was able to directly identify STs using uncorrected long reads within 10 min for all of the 96 barcoded samples simultaneously. This thus indicates that our proposed protocol can execute molecular typing for 96 multiplexed PCR amplicons within 8 h. Table 2 presents the results obtained after executing Krocus on 4,000 reads per sample (as detailed in Supplementary Table). Among the 480 sequencing samples, Krocus predicted 454 STs with >99% coverage and labeled only 26 samples as “ND,” indicating that they were untypable due to (1) low sequencing depth of genes, (2) novel combinations of alleles, or (3) new alleles. Of the 26 samples labeled as ND, 13 with <99% coverage had previously been labeled as LSD. Because a total of 352 alleles had been previously Sanger sequenced (Liou et al., 2020), and these alleles (50 × 7 + 2) were conducted separately in Run2 and Run4. Among the 704 alleles (352 × 2) subjected to Sanger sequencing, all were correctly predicted by Krocus, except one allele in barcode46 in Run2 was wrongly predicted to be *tpi*_58 rather than *tpi*_26. Krocus incorrectly predicted this sample (barcode46, ST398) to be ND with 100% coverage. Nevertheless, MinION nanopore sequencing coupled with Krocus provided a 98.86% accuracy (703/704 = 99.86%) in allele typing. In addition, Krocus predicted two other samples to be ND with 100% coverage in Run2 and in Run4 (Table 2); this could be attributed to a submission of new alleles (*pta*_664 and *glpF*_732; Liou et al., 2020) without corresponding ST information to PubMLST (Jolley et al., 2018). Finally, of the 26 samples labeled as ND, the other 13 were predicted by Krocus to be ND with ≧99% coverage; these samples were further evaluated to identify the presence of either a novel combination of alleles or new alleles (Table 3).

**Table 2:** Krocus sequence type (ST) prediction for *Staphylococcus aureus*.

Krocus result		Number of samples^2^				
Coverage	Prediction^1^	Run1	Run2	Run3	Run4	Run5
100	ST	83	91	89	91	85
≧99	ST	8	1	2	2	2
100	ND	2	3	3	2	1
≧99	ND	0	0	0	0	2
<99	ND	**3**	**1**	**2**	**1**	**6**

1 ST: sequence type predicted by Krocus; ND: sequence type not determined by Krocus.

2 Boldface indicates the number of samples labeled as low sequencing depth (LSD).

**Table 3:** Alleles prediction inconsistencies between Krocus and nanoMLST2.

Run	BC	Krocus			nanoMLST2^2^	Sanger^3^
		ST	Cov	Allele^1^		
1	03	15	99.68	*arcC*(13)*	**New allele**	*arcC*_826
1	16	623	99.65	*pta*(4)*	**New allele**	*pta*_842
1	32	7	99.71	*tpi*(6)*	**New allele**	*tpi*_786
1	36	59	100	*arcC*(19)	**New allele**	*arcC*_834
1	43	ND	100	*glpF*(19), *tpi*(58)	***glpF*_344, *tpi*_26**	*glpF*_344, *tpi*_26
1	59	1	99.71	*tpi*(1)*	**New allele**	*tpi*_787
1	65	25	99.71	*glpF*(4)*	**New allele**	*glpF*_890
1	70	22	99.77	*aroE*(6)*	**New allele**	*aroE*_1016
1	72	239	99.77	*arcC*(2)*	**New allele**	*arcC*_820
1	93	ND	100		New ST	
1	94	5,535	99.87	*gmk*(438)*	**New allele**	*gmk*_554
2	46	ND	100	*tpi*(58)	***tpi*_26**	*tpi*_26^#^
2	77	ND	100	***pta*(664)**	**New ST**	*pta*_664^#^
2	89	ND	100	***glpF*(732)**	**New ST**	*glpF*_732^#^
3	13	59	100	*tpi*(20)	**New allele**	*tpi*_781
3	29	188	100	*tpi*(1)	**New allele**	*tpi*_790
3	39	398	100	*glpF*(19)	***glpF*_344**	*glpF*_344
3	41	ND	100		New ST	
3	42	ND	100	*tpi*(58)	***tpi*_26**	*tpi*_26
3	48	291	100	*tpi*(26)	**New allele**	*tpi*_791
3	51	ND	100	*tpi*(58)	***tpi*_26**	*tpi*_26
3	80	4,567	99.65	*aroE*(35)*	**New allele**	*aroE*_1022
4	01	ND	100	***glpF*(732)**	**New ST**	*glpF*_732^#^
4	85	ND	100	***pta*(664)**	**New ST**	*pta*_664^#^
5	04	188	99.68	*pta*(1)*	**New allele**	*pta*_844
5	35	ND	99.94	*tpi*(58)*	***tpi*_26**	*tpi*_26
5	36	ND	100	*tpi*(58)	***tpi*_26**	*tpi*_26
5	37	ND	99.74	*glpF*(213)*	**New allele**	*glpF*_891
5	59	7	100	*arcC*(5)	**New allele**	*arcC*_830

1 Asterisks represent alleles partially covered using Krocus (Page and Keane, 2018), strikethroughs represent wrong predictions, and boldface represents allele prediction consistency between Krocus and Sanger sequencing.

2 Boldface represents consistency in consensus sequences between nanoMLST2 and Sanger sequencing.

3 Underlines represent new Sanger-sequenced alleles identified in this study, and # represents alleles that were Sanger sequenced previously (Liou et al., 2020).

### New Allele Types Identified by NanoMLST2

The sequencing reads of 467 samples, excluding those labeled LSD, were analyzed for consensus sequence generation and MLST typing using nanoMLST2. The results obtained through nanoMLST2 were similar to those obtained using Krocus, and 100% accuracy was observed in the 704 Sanger-sequenced alleles in Run2 and Run4 (Supplementary Table). This perfect value not only suggests the accuracy of nanoMLST2, but also means no effects of the carryover reads on the MLST typing of the following runs. Through a comparison of the results obtained using Krocus and nanoMLST2, 29 samples containing 24 alleles were identified to be inconsistent between the methods (Table 3). Nevertheless, nanoMLST2 had exceptionally good agreement with Krocus (3,245/3,269 = 99.27%). The inconsistent alleles were later sequenced using Sanger. As indicated in Table 3, the 16 new alleles identified through nanoMLST2 were all validated using Sanger sequencing to ensure accuracy. In addition, the consensus sequences generated by nanoMLST2 were full-length genes ranging from 1,067 to 1,489 bp.

Krocus was used to predict *S. aureus* STs directly from uncorrected reads (Page and Keane, 2018). However, in addition to the expected predictive failures in 22 samples (Table 3) with 16 new alleles and 6 new STs, Krocus wrongly classified eight alleles (indicated by strikethroughs) in seven samples. As listed in Table 3, Krocus frequently predicted *glpF*_344 to *glpF*_19 and *tpi*_26 to *tpi*_58, while these two pairs differ by one nucleotide. In contrast to Krocus, nanoMLST2 could generate consensus sequences for new alleles, and all of the sequences were later validated through Sanger. Therefore, the findings of this study indicate that the benefits gained through consensus sequence generation of MinION nanopore sequencing may address the needs of a wide range of kilobase-long molecular typing.

### Capacity of MinION for Routine Molecular Typing

In this study, more than 3,000 kilobase-long consensus sequences conducted in five batches (Run1 to Run5) were obtained using a single MinION flow cell. Although the flow cell was washed and reused for the successive runs (Run2 to Run5), the variation between the STs in the different batches indicates that the effects of the cell reuse were marginal. For example, the STs of barcode10 in Run1, Run2, Run3, Run4, and Run5 were 15, 6, 188, 254, and 59, respectively. However, consistent STs were obtained through Sanger (STs of 6 and 254 in Run2 and Run4), demonstrating that the STs were not influenced by the preceding runs. Regarding rapid and routine molecular typing, the results of the first three runs of the 96 samples (Run1 to Run3) were obtained 4–6 h after the initiation of the sequencing process (3.6–5 h run time plus 0.5 h data analysis; Table 1). The remaining two runs (Run4 and Run5) required a longer sequencing period because of the reduced number of available pores (<400) in the used MinION; nevertheless, the generated consensus sequences remained accurate for routine molecular typing. Another flow cell containing more than 1,500 available pores in the beginning was also used in this study, shorter sequencing periods (1.6–3.5 h) were required for the five runs. As shown in Figure 2, the aliquots of amplicons from the first flow cell Run2, Run3, Run5, Run1, and Run3 were used in the second flow cell Run1–Run5, respectively. Although the sequencing components in the preceding run varied, all the STs obtained from the second flow cell were identical to that of the corresponding samples in the first flow cell (Supplementary Table), which again reveals that the effects of the cell reuse were marginal and the STs were not influenced by the preceding runs.

Compared with the approach used in our previous study (Liou et al., 2020), the proposed protocol in this study not only reduced the primer cost significantly but also reduced the PCR reagents and amounts of manual effort by seven times. Library preparation in this protocol cost US$110 for a total of 96 samples; by contrast, the cost incurred by ligation-based procedures (Liou et al., 2020) for such a run is US$148, regardless of whether third-party consumables such as AmpureXP beads and NEB End Repair/dA-Tailing enzymes are required (Liou et al., 2020). The MinION flow cell used in our protocol could produce accurate typing results for a total of 3,269 kilobase-long genes, in addition to affording less expensive and more rapid multiplexing PCR and library preparation. This protocol allows for a more efficient and cost-effective method for routine molecular typing at an estimated cost of US$4 per sample (Supplementary Table). Specifically, through the protocol, the cost of a kilobase-long gene would be less than US$1, which is substantially less than that in Sanger sequencing. Overall, our results demonstrate the effectiveness of the portable MinION sequencer in providing accurate, rapid, and routine molecular typing.

## Conclusion

The features of friendly access (USD$1,000 for a starter pack), portability and the ability to monitor real-time output and reuse of a flow cell of the ONT MinION sequencer remove the barriers of accessing accurate, rapid, and routine molecular typing in small- to medium-sized laboratories. To the best of our knowledge, our study is the first to investigate the reusability of a MinION flow cell and to provide the evidence of the sequencing accuracy of a reused flow cell. In this study, a workflow was designed entailing the use of one universal primer set with seven primer pairs of housekeeping genes to amplify full-length target genes simultaneously for *S. aureus* MLST and rapid barcoding in conjunction with ONT nanopore sequencing on a portable MinION platform followed by the nanoMLST2 analysis. Our results indicate that the benefits gained through consensus sequence generation using this workflow may address the needs of a wide range of kilobase-long molecular typing.

## Data Availability Statement

The datasets presented in this study can be found in online repositories. The names of the repository/repositories and accession number(s) can be found below:

https://doi.org/10.6084/m9.figshare.19179107

https://doi.org/10.6084/m9.figshare.19179110

https://doi.org/10.6084/m9.figshare.19179116

https://doi.org/10.6084/m9.figshare.19179122

https://doi.org/10.6084/m9.figshare.19179125

https://doi.org/10.6084/m9.figshare.19179128.

## Author Contributions

Y-CL, H-CW, and F-JC conceived the study. Y-CL and H-CW designed the methodology. Y-CL implemented the pipeline. H-CW and C-HL validated data. Y-CL, H-CW, C-HL, and F-JC investigated. T-LL, I-WH, and J-FL provided the strains and participated in discussion of the study. Y-CL, H-CW, T-LL, and F-JC wrote the manuscript. Y-CL and F-JC supervised and acquired funding. All authors contributed to the article and approved the submitted version.

## Funding

This work was supported by intramural grants from National Health Research Institutes (IV-110-PP-06 to F-JC and PH-110-PP-05 to Y-CL) and Ministry of Science and Technology (MOST 110-2314-B-400-038).

## Conflict of Interest

The authors declare that the research was conducted in the absence of any commercial or financial relationships that could be construed as a potential conflict of interest.

## Publisher’s Note

All claims expressed in this article are solely those of the authors and do not necessarily represent those of their affiliated organizations, or those of the publisher, the editors and the reviewers. Any product that may be evaluated in this article, or claim that may be made by its manufacturer, is not guaranteed or endorsed by the publisher.

## Footnotes

^1^ https://github.com/nanoporetech/medaka
